# Genome sequences of *Lactiplantibacillus pentosus* isolated from Spanish and Greek table olive fermentations

**DOI:** 10.1128/mra.01358-25

**Published:** 2026-02-23

**Authors:** Elio López-Garcí­a, Antonio Benítez-Cabello, Ilenys M. Pérez-Díaz, Efstathios Z. Panagou, José Luis Ruíz-Barba, Eduardo Medina, Ana Marín-Gordillo, Francisco Noé Arroyo-López

**Affiliations:** 1 Food Biotechnology Department, Instituto de la Grasa (CSIC), Universitario Pablo de Olavide 54444, Seville, Spain; 2 USDA-Agricultural Research Service, Southeast Area, Food Science & Market Quality and Handling Research Unit 57786 , Raleigh, North Carolina, USA; 3 Laboratory of Microbiology and Biotechnology of Foods, Department of Food Science and Human Nutrition, School of Food and Nutritional Sciences, Agricultural University of Athens 68995, Athens, Greece; 4 Oleica start-up (Technological Applications for Improvement of Quality and Safety in Foods), Seville, Spain; Nanchang University, Nanchang, Jiangxi, China

**Keywords:** whole-genome sequencing, olive fermentation, food microbiology, *Lactobacillus*

## Abstract

We report 10 assembled and annotated whole-genome sequences of *Lactiplantibacillus pentosus* isolated from Greek (*n* = 3) and Spanish (*n* = 7) commercial table olive fermentations. The number of contigs per genome sequence fluctuates between 9 and 192.

## ANNOUNCEMENT

Table olives are among the most important fermented vegetables in the Mediterranean Basin, with a global production exceeding 3 million tons per year. The main producing countries include Spain, Egypt, Turkey, Greece, and Italy (1). *Lactiplantibacillus pentosus* (*Lp. pentosus*) is among the predominant lactic acid bacteria species responsible for table olive fermentation, contributing to the flavor, quality, and safety of the final product (2). Ten *Lactiplantibacillus pentosus* colonies were isolated from distinct table olive fermentations conducted in Spain or Greece (Table 1). All isolates were obtained from table olive fermentation that had been spiral plated on Lactobacillus de Man-Rogosa-Sharpe (MRS) agar supplemented with 0.02% sodium azide (Sigma, St. Louis, MO, USA) and incubated at 37°C. The isolates *Lp. pentosus* B281, *Lp. pentosus* B282, and *Lp. pentosus* B356 were obtained from natural black olive fermentation of Greece. On the other hand, the isolate *Lp. pentosus* L190 was obtained from natural green table olives from Spain. Finally, the remaining isolates were obtained from fermentations of Spanish-style (lye-treated) green table olives. Molecular identification of isolates was performed by sequencing the 16S rDNA gene using the oligonucleotide pairs 27F/1492R. The percentage of identity of the sequences was determined through a BLAST analysis with the available sequences from the NCBI GenBank database. Since 16S rDNA sequence analysis could not differentiate at species level within *Lactiplantibacillus plantarum* group, a multiplex PCR of the recA gene was carried out to discriminate between *Lp. pentosus*, *Lp. plantarum*, and *Lp. paraplantarum* species (3). Prior to DNA extraction, the strains were grown on Lactobacillus MRS broth (Oxoid, Basingstoke, U.K.) at 37°C for 48 h. Genomic and plasmid DNA was extracted and purified as described previously by Martín-Platero et al. (4). Briefly, DNA was extracted using a modified salting-out procedure consisting of three steps: resuspension of cells in enzymatic buffer, bacterial lysis in the corresponding buffer for 5 min at 80°C, and protein precipitation by adding 5 M sodium chloride. The integrity of the DNA was confirmed via agarose gel electrophoresis (0.9%), and its concentration was determined using a Qubit 4 fluorometer (Thermo Fisher Scientific, Waltham, USA). The DNA extracted from the 10 *Lp. pentosus* isolates was sequenced using an Illumina MiSeq platform (FISABIO, Valencia, Spain) with libraries prepared using the Illumina Nextera DNA Flex Library Prep Kit, generating paired-end reads of 2 × 300 bp. In addition, *Lp. pentosus* LP309 was sequenced using Oxford Nanopore Technologies on a MinION device (Instituto de la Grasa, Seville, Spain) using the Ligation Sequencing Kit V14 (SQK-LSK114) (Oxford Nanopore Technologies, Didcot, UK) and R10.4.1 flow cells, following the manufacturer’s standard protocols. No DNA shearing or size selection was performed prior to library preparation using the ligation sequencing kit. Basecalling of Nanopore reads was conducted using super-accurate mode. Raw sequence reads were quality-trimmed using TrimGalore version 0.6.10 (5) for Illumina data and Porechop version 0.2.4 (6) for Nanopore reads from *Lp. pentosus* LP309. Read quality was subsequently assessed using FastQC version 0.11.9 (7) and Nanoplot version 1.44.1 (8), respectively. The trimming was with a quality of higher than 30 to Illumina reads and 10 to ONT reads. Moreover, the reads originating from ONT were removed with length less than 500 bp. Genome assembly was performed using Unicycler version 0.5.1 (9), with parameters for minimum length to contig as 300 bp; the other parameters were maintained as standard. For isolate LP309, it was assembled with hybrid assembly using Illumina and ONT reads, and then the genome was closed through Unicycler software during assembly as default parameters. Structural and functional annotation of the newly sequenced genomes was carried out using the Prokaryotic Genome Annotation System (Prokka) version 1.14.6 (10) with default parameters. The raw reads and annotated genomes were deposited in the European Nucleotide Archive under the BioProject accession number PRJEB94968, with the individual accession numbers provided in Table 1.

**TABLE 1:** Accession numbers, assembly size, number of contigs, estimated coverage, read pairs, GC content, N50, and isolates' site of collection corresponding to the *Lactiplantibacillus pentosus* genome sequences described in this announcement

Strain ID	BioSample accession no.	SRA accession no.	Assembly size (bp)	Contigs	Estimated coverage (×)	No. of reads	Avg raw read length (bp)	GC %	N50 (bp)	Isolate collection site	Yr of isolation
*Lactiplantibacillus pentosus* B281	SAMEA118838861	ERS25357728	3,898,496	196	78.076	1,323,868	117.2	45.90	78,159	Black Olive Fermentations, Greece	2012
*Lactiplantibacillus pentosus* B356	SAMEA118838862	ERS25357729	3,855,511	171	74.905	1,298,196	113.0	45.87	100,349	Black Olive Fermentations, Greece	2012
*Lactiplantibacillus pentosus* B371	SAMEA118838863	ERS25357730	3,785,466	74	80.494	1,575,650	97.5	46.01	239,262	Black Olive Fermentations, Greece	2012
*Lactiplantibacillus pentosus* L190	SAMEA118838866	ERS25357733	3,780,014	80	82.439	1,337,036	117.8	46.03	123,041	Natural Table Olive Fermentation, Spain	2010
*Lactiplantibacillus pentosus* AdC7	SAMEA118838860	ERS25357727	3,838,926	49	72.515	1,487,318	94.3	45.98	428,723	Lye-treated Table Olive Fermentation, Spain	2010
*Lactiplantibacillus pentosus* F3.3	SAMEA118838864	ERS25357731	3,671,544	182	71.816	1,268,094	105.9	46.13	57,125	Lye-treated Table Olive Fermentation, Spain	2013
*Lactiplantibacillus pentosus* J41.1	SAMEA118838865	ERS25357732	3,673,301	200	75.147	1,282,936	110.0	46.04	40,567	Lye-treated Table Olive Fermentation, Spain	2010
*Lactiplantibacillus pentosus* LP309	SAMEA118838869	ERS25357736 ERS27177130	3,743,270	9	1,031.79	10,748,663 (Illumina); 234,372 (ONT)	149.5 (Illumina); 2,764.2 (ONT)	46.12	3,523,074	Lye-treated Table Olive Fermentation, Spain	2019
*Lactiplantibacillus pentosus* L119	SAMEA118838867	ERS25357734	3,763,867	132	141.404	3,597,628	74.5	46.08	80,048	Biofilms of Lye-treated Table Olive Fermentation, Spain	2017
*Lactiplantibacillus pentosus* L13B4	SAMEA118838868	ERS25357735	3,808,550	172	137.349	3,540,756	74.6	45.99	83,686	Biofilms of Lye-treated Table Olive Fermentation, Spain	2017

The genome lengths observed are consistent with previously published *Lp. pentosus* genomes available in public databases and highlight the notable plasticity and adaptability of the species (11). *Lp. pentosus* exhibits a larger average genome size (~3.7 Mb) than *Lp. plantarum* (~3.4 Mb). Over the course of evolution, members of *Lactobacillaceae* have either reduced or expanded their genomes in response to adaptation to specific ecological niches, whether host-adapted, free-living, or nomadic (11). In this context, *Lp. pentosus* has been described as a metabolically versatile species, containing a broad repertoire of genes involved in carbohydrate utilization and stress responses, which has been associated with its ability to persist across diverse habitats (12, 13).

## Data Availability

The assembled genome sequences and the corresponding raw sequencing reads are publicly available in the European Nucleotide Archive (ENA) under BioProject accession number PRJEB94968. Individual accession numbers are provided in Table 1.
